# Association of blood pressure with incident diabetic microvascular complications among diabetic patients: Longitudinal findings from the UK Biobank

**DOI:** 10.7189/jogh.13.04027

**Published:** 2023-03-24

**Authors:** Cong Li, Honghua Yu, Zhuoting Zhu, Xianwen Shang, Yu Huang, Charumathi Sabanayagam, Xiaohong Yang, Lei Liu

**Affiliations:** 1 Guangdong Eye Institute, Department of ophthalmology, Guangdong Provincial People's Hospital (Guangdong Academy of Medical Sciences), Southern Medical University, Guangzhou, China; 2 School of Medicine, South China University of Technology, Guangzhou, China; 3 Guangdong Provincial Key Laboratory of Artificial Intelligence in Medical Image Analysis and Application, Guangzhou, China; 4 Singapore Eye Research Institute (SERI) and Singapore National Eye Centre, Population Health, Singapore, Singapore; 5 Duke-NUS Medical School, Singapore, Singapore; 6 National University of Singapore, Singapore, Singapore; 7 Department of Ophthalmology, Jincheng People's Hospital, Jincheng, Shanxi Province, China

## Abstract

**Background:**

Evidence suggests a correlation of blood pressure (BP) level with presence of diabetic microvascular complications (DMCs), but the effect of BP on DMCs incidence is not well-established. We aimed to explore the associations between BP and DMCs (diabetic retinopathy, diabetic kidney disease, and diabetic neuropathy) risk in participants with diabetes.

**Methods:**

This study included 23 030 participants, free of any DMCs at baseline, from the UK Biobank. We applied multivariable-adjusted Cox regression models to estimate BP-DMCs association and constructed BP genetic risk scores (GRSs) to test their association with DMCs phenotypes. Differences in incidences of DMCs were also compared between the 2017 ACC/AHA and JNC 7 guidelines (traditional criteria) of hypertension.

**Results:**

Compared to systolic blood pressure (SBP)<120 mm Hg, participants with SBP≥160 mm Hg had a hazard ratio (HR) of 1.50 (95% confidence interval (CI) = 1.09, 2.06) for DMCs. Similarly, DMCs risk increased by 9% for every 10 mm Hg of higher SBP at baseline (95% CI = 1.04, 1.13). The highest tercile SBP GRS was associated with 32% higher DMCs risk (95% CI = 1.11, 1.56) compared to the lowest tercile. We found no significant differences in DMCs incidence between JNC 7 and 2017 ACC/AHA guidelines.

**Conclusions:**

Genetic and epidemiological evidence suggests participants with higher SBP had an increased risk of DMCs, but hypertension defined by 2017 ACC/AHA guidelines may not impact DMCs incidence compared with JNC 7 criteria, contributing to the care and prevention of DMCs.

Diabetic microvascular complications (DMCs) characterised by diabetic retinopathy (DR), diabetic kidney disease (DKD), and diabetic neuropathy (DN), are highly prevalent among populations with diabetes. Meta-analyses based on clinical trials suggested that lowering blood pressure (BP) can effectively reduce the risk of diabetes and its microvascular and macrovascular complications [1,2]. In the past few years, the UK Prospective Diabetes Study (UKPDS) showed that the incidence of clinical DMCs (predominantly retinal photocoagulation) was significantly associated with raised systolic BP (SBP) [3]. Moreover, several previous studies evaluated the associations between high BP variability and adverse microvascular outcomes in patients with type 2 diabetes [4-10]. However, most of them focused only on nephropathy [5-7,10], with inconclusive outcomes for retinopathy [8,9]. Furthermore, a long-term prospective cohort study addressing the influence of BP on all components of DMCs is still needed, as this is an essential strategy for disease prevention.

Notably, compared to the phenotype, the genetic risk score (GRS) represents the congenital risk of a disease, which is less influenced by environmental or other systemic confounding factors. Hence, investigating a disease-trait association from both phenotypic and genotypic aspects is now widely accepted [11,12]. However, the association between BP GRSs and DMCs remains unclear.

There is vast evidence on the role of hypertension in the occurrence and progression of DMCs [13,14]. In 2017, the American College of Cardiology (ACC) and the American Heart Association (AHA) released an updated guideline, which changed the definition of hypertension, lowering the cut-off for defining hypertension to SBP/diastolic blood pressure (DBP)≥130/80 mm Hg [15], while the upper end of pre-hypertension based on the seventh report of the Joint National Committee (JNC 7) [16] was reclassified as stage 1 hypertension. In contrast to the updated American guideline, the 2018 European Society of Cardiology (ESC)/European Society of Hypertension (ESH) BP guideline defined hypertension based on a threshold of ≥140/90 mm Hg [17], which is the same as for JNC 7. With the recent guidelines gradually becoming popular, exploring the impacts of stricter definitions for related disease prevalence, treatment, and control to reduce the disease burden worldwide is crucial. However, no report has determined the difference in DMCs incidence using the 2017 ACC/AHA rule, and compared this result with the JNC 7 rule.

We used data from a large-scale UK Biobank population-based cohort over more than 10 years of follow-up, in order to examine the association between BP levels and incidence of DMCs, investigate the associations of BP GRSs with longitudinal DMCs, and determine any differences in incidence of DMCs in relation to hypertension, according to the JNC 7 and 2017 ACC/AHA guidelines.

## METHODS

### Study design and population

We used data from the UK Biobank, which is available from a public, open-access repository; its profile and detailed methods have been described elsewhere [18]. Briefly, a total of 502 628 participants (aged 40-69 years) from the general population were recruited between 2006 and 2010 at one of 22 assessment centres in Scotland, England, or Wales.

**Figure 1:**
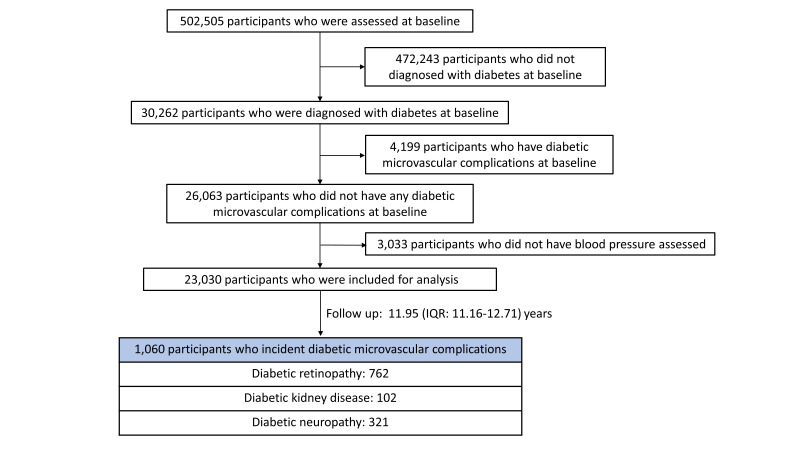
Flowchart for population selection from UK Biobank.

We included 30 262 individuals with diabetes at recruitment. Diabetes cases were defined as those who had self-reported or doctor-diagnosed diabetes mellitus, were taking anti-hyperglycaemic medications or using insulin, or glycosylated haemoglobin (HbA1c)>48 mmol/mol [19]. Finally, we included data from 23 030 participants with diabetes in the main analysis after excluding participants with any type of DMCs at baseline (4199) or missing values on BP (3033) (**Figure 1**).

### Blood pressure measurement

Trained nurses measured BP (mmHg) twice at one-minute intervals using a digital sphygmomanometer (Omron 705 IT; OMRON Healthcare Europe B.V., Hoofddorp, Netherlands) after the participant had been at rest for at least five minutes in the seated position. We used the average of the two repeated measurements in the analysis. We diagnosed hypertension using two different guidelines: the JNC 7 (SBP/DBP≥140/90 mm Hg) and the 2017 ACC/AHA (SBP/DBP≥130/80 mm Hg) (Table S1 in the **Online Supplementary Document**). Furthermore, hypertension was classified into three categories: isolated diastolic hypertension (IDH) (SBP<140 mm Hg and DBP≥90 mm Hg for JNC 7; SBP<130 mm Hg and DBP≥80 mm Hg for 2017 ACC/AHA), isolated systolic hypertension (ISH) (SBP≥140 mm Hg and DBP<90 mm Hg for JNC 7; SBP≥130 mm Hg and DBP<80 mm Hg for 2017 ACC/AHA), and systolic-diastolic hypertension (SDH) (SBP≥140 mm Hg and DBP≥90 mm Hg for JNC 7; SBP≥130 mm Hg and DBP≥80 mm Hg for 2017 ACC/AHA) [20].

### Ascertaining incident diabetic microvascular complication cases

We identified the DMCs (DR, DKD, and DN) using the algorithms provided by UK Biobank, which were based on self-reported and data from electronic health records according to ICD-9 and ICD-10 codes (Table S2 in the **Online Supplementary Document**). Our analysis of DMCs incidence excluded individuals with any diagnosed or self-reported DMCs prior to the baseline assessment. We calculated the follow-up time as the duration between the date of baseline assessment and censored at the date of DMCs incidence, date of death, date lost to follow-up, or the end of follow-up, whichever occurred first.

### Data collection and assessment of covariates

Extensive phenotypic and genotypic data were collected at recruitment. All participants completed touch-screen questionnaires collecting information on socio-demographics, habitual diet, lifestyle factors, and medical history, underwent physical examinations on anthropometric measurements, and provided biological samples including of blood, urine, and saliva.

The confounders in our analysis included age, sex, body mass index, Townsend index (an area-based proxy measure for socioeconomic status), smoking status (recorded as current/previous and never), alcohol assumption (recorded as current/previous and never), HbA1c, duration of diabetes, use of anti-hyperglycaemic and anti-hypertension medication, estimated glomerular filtration rate (eGFR), total cholesterol (TC), high-density lipoprotein cholesterol (HDL-C), low-density lipoprotein cholesterol (LDL-C), and triglycerides (TG).

### Genetic risk score for blood pressure

BiLEVE Axiom array, or the UK Biobank Axiom array was used for genotyping by Affymetrix, and approximately 450 000 UK Biobank participants were genotyped. The genotype imputation using the Haplotype Reference Consortium reference panel was conducted by the UK Biobank researchers before data were released, followed by extensive quality control [21].

Genetic variants associated with BP were selected based on a recent genome-wide association study conducted among over one million people of European ancestry by Evangelou et al [22]. Following their method, a total number of 885 single nucleotide polymorphisms (SNPs) associated with BP were used for GRS calculation. For each SNP, the magnitude of its association (beta coefficient) with BP was used as the weighting factor and, for each participant, the dosage of the risk allele times the weight was calculated, and the sum across all the SNPs was considered as the GRS. In PLINK 2.0, the–score function was used for GRS generation.



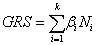



The above formula was used to generate GRS, where k is the number of independent genetic variants associated with BP, β*_i_* is the effect estimate, and *N_i_* is the number of risk alleles for each locus.

### Ethics

The UK Biobank study was previously given ethical approval by the National Information Governance Board for Health and Social Care and the NHS North West Multicentre Research Ethics Committee (11/NW/0382). All participants completed written informed consent before enrolment. This study was conducted under application number 62489 of the UK Biobank resource.

### Statistical analysis

We reported the data as mean and standard deviation (SD) for normally distributed variables, median and interquartile range (IQR) for skewed variables, and number and percentage for categorical variables. We compared the baseline characteristics of participants from UK Biobank of groups with and without DMCs by Mann-Whitney or unpaired *t*-tests for continuous data, and Pearson χ^2^ or Fisher exact tests for categorical data. To indicate the relationship between BP and the risk of DMCs incidence, we modelled BP as restricted quadratic splines to provide a smooth, yet flexible description of the dose-response relationship. We estimated the hazard ratios (HRs) and 95% confidence intervals (CIs) of DMCs among different levels and categories of BP by Cox proportional hazards model. We conducted subgroup analyses to test whether the association between BP and DMCs incidence differed between groups of sex, HbA1c, and use of anti-hyperglycaemic and anti-hypertensive medication. Furthermore, we tested how the BP GRSs were associated with DMCs risk by estimating HR and 95% CIs across GRS terciles (i.e., top and bottom terciles represent high and low genetic risk, respectively); we examined linear trends using the GRS as a continuous variable. Additionally, we calculated the incidence rate with 95% CI of diabetic adults with newly diagnosed DMCs based respectively on the 2017 ACC/AHA and the JNC 7 guidelines.

We performed statistical analyses with Stata version 16 (StataCorp LLC, College Station, Texas USA) and R software (www.r-project.org, version 4.1.2). We calculated the *P*-value for trends for BP categories. We imputed missing data using multiple imputation by chained equations in Stata. All *P* values were two-sided with significance set at *P* < 0.05.

## RESULTS

### Baseline characteristics

We included 23 030 individuals (median age 61.00 years, 40.08% females) for the final analysis. Over the mean 11.95 (IQR = 11.16-12.71) years of the follow-up period, a total of 1060 (4.60%; 95% CI = 4.34%, 4.88%) diabetic individuals developed DMCs, of which 762 (3.31%; 95% CI = 3.08%, 3.55%) suffered from DR, 102 (0.44%; 95% CI = 0.36%, 0.54%) suffered from DKD, and 321 (1.39%; 95% CI = 1.25%, 1.55%) suffered from DN. The clinical characteristics of the participants with and without incident DMCs are shown in **Table 1**. Participants with incident DMCs were mostly older, of male sex, used anti-hyperglycaemic medication, and had higher BMI, higher Townsend index, longer diabetic duration, higher SBP, and higher HbA1c levels, but lower DBP, TC, LDL-C, and were more often current/former drinkers than participants without incident DMCs.

**Table 1:** Characteristics of participants with and without incident diabetic microvascular complications*

Variables	Total	With microvascular complications	Without microvascular complications	*P*-value*
**N**	23 030	1 060	21 970	
**Age in years, median (IQR)**	61.00 (54.00, 65.00)	62.00 (55.00, 66.00)	61.00 (54.00, 65.00)	<0.001
**Sex, n (%)**				0.007
Females	9230 (40.08)	383 (36.13)	8847 (40.27)	
Males	13 800 (59.92)	677 (63.87)	13 123 (59.73)	
**Ethnicity, n (%)**				0.938
White	19 883 (86.34)	916 (86.42)	18 967 (86.33)	
Others	3147 (13.66)	144 (13.58)	3003 (13.67)	
**BMI in kg/m^2^, mean (SD)**	31.32 (5.77)	31.94 (5.93)	31.29 (5.77)	<0.001
**Townsend index, median (IQR)**	-1.32 (-3.21, 1.93)	-0.80 (-3.07, 2.78)	-1.34 (-3.22, 1.88)	<0.001
**Education level, n (%)**				0.385
College or university degree	17472 (75.87)	816 (76.98)	16 656 (75.81)	
Others	5558 (24.13)	244 (23.02)	5314 (24.19)	
**Smoking status, mean (%)**				0.163
Never	10 736 (46.62)	472 (44.53)	10 264 (46.72)	
Former/current	12 294 (53.38)	588 (55.47)	11 706 (53.28)	
**Alcohol consumption**, **mean (%)**				0.017
Never	2089 (9.07)	118 (11.13)	1971(8.97)	
Former/current	20 941 (90.93)	942 (88.87)	19 999 (91.03)	
Duration of diabetes in years, median (IQR)	5.00 (2.17, 9.00)	8.00 (4.00, 14.00)	5.00 (2.00, 8.80)	<0.001
SBP in mmHg, mean (SD)	141.47 (17.18)	143.43 (18.04)	141.37 (17.13)	<0.001
DBP in mmHg, mean (SD)	82.53 (9.34)	81.57 (10.01)	82.57 (9.31)	0.001
HbA1c in mmol/mol, mean (SD)	52.64 (14.70)	60.22 (17.50)	52.28 (14.40)	<0.001
TG in mmol/L, median (IQR)	1.89 (1.31, 2.70)	1.94 (1.32, 2.75)	1.88 (1.31, 2.70)	0.294
TC in mmol/L, mean (SD)	4.71 (1.14)	4.52 (1.09)	4.72 (1.15)	<0.001
LDL-C in mmol/L, mean (SD)	2.86 (0.86)	2.71 (0.80)	2.86 (0.85)	<0.001
HDL-C in mmol/L, mean (SD)	1.21 (0.32)	1.19 (0.35)	1.21 (0.32)	0.107
eGFR in mL/(min ×1.73 m^2^), median (IQR)	93.78 (84.15, 100.76)	93.30 (83.03, 100.95)	93.81 (84.20, 100.75)	0.370
**Anti-hyperglycaemic medication, n (%)**				<0.001
No	9724 (42.22)	188 (17.74)	9536 (43.40)	
Yes	13 306 (57.78)	872 (82.26)	12 434 (56.60)	
**Anti-hypertension medication, n (%)**				0.354
No	18 249 (79.24)	828 (78.11)	17 421 (79.29)	
Yes	4781 (20.67)	232 (21.89)	4549 (20.71)	

SD – standard deviation, IQR – interquartile range, BMI – body mass index, SBP – systolic blood pressure, DBP – diastolic blood pressure, HbA1c – glycosylated haemoglobin, TG – triglycerides, TC – total cholesterol, LDL-C – low-density lipoprotein cholesterol, HDL-C – high-density lipoprotein cholesterol, eGFR – estimated glomerular filtration rate

*Values with *P* < 0.05 are considered as statistically significant for the differences between the two groups.

### Blood pressure and diabetic microvascular complications

The frequency distribution of DMCs for different BP levels is shown in **Table 2**. The trends for incidence of overall DMCs, DR, and DKD significantly rose with increasing SBP, but not for individuals with DN. With the increase of DBP, the change trends for incidence of overall DMCs and DR were significant, but not for DKD and DN.

**Table 2:** Frequency distribution of diabetic microvascular complications by different blood pressure levels

Blood pressure level (mmHg)	Overall		DR		DKD		DN	
	**No**	**Incident (95% CI)**	**No**	**Incident (95% CI)**	**No**	**Incident (95% CI)**	**No**	**Incident (95% CI)**
**Systolic BP**								
<120	83	4.05 (3.24, 5.00)	54	2.64 (1.99, 3.43)	9	0.44 (0.20, 0.83)	28	1.37 (0.91, 1.97)
120-129	155	4.05 (3.45, 4.72)	111	2.90 (2.39, 3.48)	8	0.21 (0.09, 0.41)	51	1.33 (0.99, 1.75)
130-139	247	4.60 (4.05, 5.19)	162	3.01 (2.57, 3.51)	18	0.33 (0.24, 0.61)	89	1.66 (1.33, 2.03)
140-149	215	4.28 (3.74, 4.88)	161	3.20 (2.74, 3.73)	20	0.40 (0.24, 0.61)	59	1.17 (0.90, 1.51)
150-159	182	5.17 (4.47, 5.96)	142	4.04 (3.42, 4.82)	19	0.54 (0.33, 0.84)	48	1.36 (1.01, 1.81)
≥160	178	5.50 (4.74, 6.34)	132	4.08 (3.42, 4.82)	28	0.86 (0.58, 1.25)	46	1.42 (1.04, 1.89)
*P*-value for trend*	0.001		<0.001		<0.001		0.794	
**Diastolic BP**								
<80	474	5.18 (4.73, 5.65)	346	3.78 (3.40, 4.19)	43	0.47 (0.34, 0.63)	137	1.50 (1.26, 1.77)
80-89	385	4.38 (3.96, 4.83)	269	3.06 (2.71, 3.44)	36	0.41 (0.29, 0.57)	122	1.39 (1.15, 1.66)
90-99	158	3.77 (3.21, 4.39)	113	2.70 (2.23, 3.23)	17	0.41 (0.24, 0.65)	50	1.19 (0.89, 1.57)
≥100	43	4.81 (3.50, 6.42)	34	3.80 (2.65, 5.27)	6	0.67 (0.25, 1.46)	12	1.34 (0.70, 2.33)
*P*-value for trend*	0.002		0.009		0.993		0.214	

CI – confidence interval, DR – diabetic retinopathy, DKD – diabetic kidney disease, DN – diabetic neuropathy, BP – blood pressure

*Values with *P* < 0.05 are considered statistically significant.

Cox proportional hazards models were used to investigate the relationship between BP and incident DMCs (**Table 3**). Compared to participants with SBP<120 mm Hg, the HR for DMCs was 1.50 (95% CI = 1.09, 2.06) for participants with SBP≥160 mm Hg, after adjusting for confounding variables. For DBP, it was not significantly associated with DMCs incidence after adjusting for confounders (all *P* > 0.05). Similarly, further analysis of BP as a continuous variable suggested that the HR for DMCs was 1.09 (95% CI = 1.04, 1.13) with every 10 mm Hg higher SBP at baseline after adjusting for covariates, but the relationship was not significant regarding the increase of DBP.

**Table 3:** Cox proportional hazards models for incident diabetic microvascular complications by different blood pressure levels*

Blood pressure level (mmHg)	Crude HR (95% CI)	*P*-value†	Adjusted HR (95% CI)	*P*-value†
**Systolic blood pressure**				
<120	Reference		Reference	
120-129	0.99 (0.76, 1.29)	0.942	1.01 (0.73, 1.38)	0.953
130-139	1.12 (0.88, 1.44)	0.364	1.21 (0.90, 1.63)	0.217
140-149	1.04 (0.81, 1.34)	0.741	1.11 (0.82, 1.51)	0.496
150-159	1.28 (0.99, 1.66)	0.064	1.33 (0.97, 1.83)	0.074
≥160	1.36 (1.05, 1.77)	0.020	1.50 (1.09, 2.06)	0.012
*P*-value for trend†	0.001		0.001	
Per 10 mm Hg higher at baseline	1.07 (1.03, 1.11)	<0.001	1.09 (1.04, 1.13)	<0.001
**Diastolic blood pressure**				
<80	Reference		Reference	
80-89	0.83 (0.73, 0.95)	0.008	0.90 (0.77, 1.05)	0.164
90-99	0.71 (0.59, 0.85)	<0.001	0.84 (0.68, 1.03)	0.089
≥100	0.92 (0.67, 1.25)	0.578	1.34 (0.96, 1.88)	0.085
*P*-value for trend†	0.001		0.600	
Per 10 mm Hg higher at baseline	0.88 (0.83, 0.94)	<0.001	0.97 (0.90, 1.05)	0.453

HR – hazard ratio, CI – confidence interval

*Adjusted for age, sex, body mass index, ethnicity, Townsend index, smoking status, alcohol consumption, glycosylated haemoglobin, low-density lipoprotein cholesterol, duration of diabetes, anti-hyperglycaemic medication, anti-hypertensive medication, and estimated glomerular filtration rate.

†Values with *P* < 0.05 are considered statistically significant.

We further used Cox proportional hazards models to determine the association between BP and incidence of DR, DKD, and DN (Table S3-S5 in the **Online Supplementary Document**, respectively). After adjusting for confounding factors, the HRs for DR were 1.73 (95% CI = 1.17, 2.56) for participants with SBP 150-159 and 1.84 (95% CI = 1.23, 2.73) for those with ≥160 mm Hg, compared with SBP<120 mm Hg. Multivariable analysis showed a higher risk of DR incidence for participants with DBP≥100 mm Hg compared with <80 mm Hg (HR = 1.60; 95% CI = 1.09, 2.35). For BP as a continuous variable, the HRs for DR and DKD were 1.12 (95% CI = 1.06, 1.17) and 1.31 (95% CI = 1.17, 1.47), respectively, with every 10 mm Hg higher SBP at baseline after adjusting for confounders, but the association was not significant for the increase of DBP.

**Figure 2:**
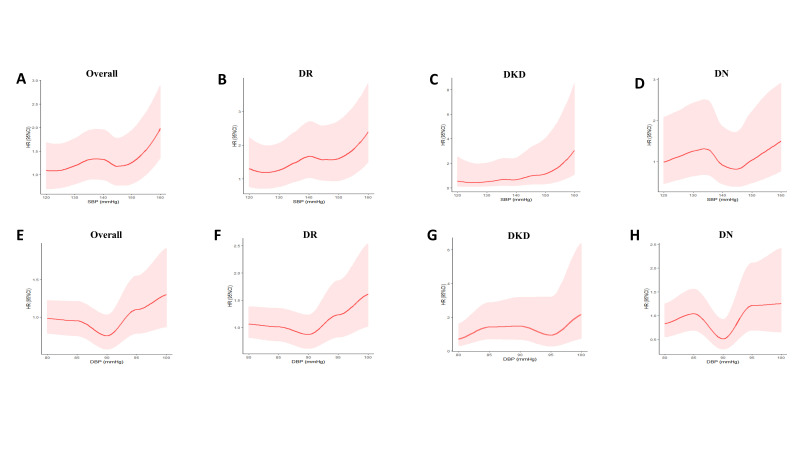
Dose-response relationship between (A-D) SBP and (E-H) DBP and diabetic microvascular complications among patients with diabetes. **Panel A.** Dose-response relationship between SBP and overall diabetic microvascular complications. **Panel B.** Dose-response relationship between SBP and DR. **Panel C.** Dose-response relationship between SBP and DKD. **Panel D.** Dose-response relationship between SBP and DN. **Panel E.** Dose-response relationship between DBP and overall diabetic microvascular complications. **Panel F.** Dose-response relationship between DBP and DRD **Panel G.** dose-response relationship between DBP and DKD. **Panel H.** Dose-response relationship between DBP and DN. DR – diabetic retinopathy, DKD – diabetic kidney disease, DN – diabetic neuropathy, SBP – systolic blood pressure, DBP – diastolic blood pressure, HR – hazard ratio, CI – confidence interval.

The SBP was associated with the risk of DMCs with a nonlinear dose-response relationship (**Figure 2**, panels A-D). Dose-response relationships between DBP and DMCs incidence among diabetes participants showed approximately J-shaped curves (**Figure 2**, panels E-H), indicating that the effect of DBP on DMCs incidence also tended to be nonlinear.

### Blood pressure GRS and diabetic microvascular complications

After adjusting for confounders, those with high genetically defined SBP (estimated mean SBP = 160 mm Hg) among the UK population showed 32% higher risk of DMCs incidence (95% CI = 1.11, 1.56) than participants at low genetic risk. With the increase of SBP genetic risk, the trends for DMCs incidence significantly increased. Similar results were found for DR and DKD incidence. However, no relationship between SBP GRS and DMCs was found when the GRS for SBP was considered a continuous variable (**Table 4**). The DBP GRS did not show a significant association with DMCs (data not shown).

**Table 4:** Cox proportional hazards models for incident diabetic microvascular complications by different systolic blood pressure GRS levels*

Systolic blood pressure GRS	Crude HR (95% CI)	*P*-value†	Adjusted HR (95% CI)	*P-*value†
**DMCs**				
Low GRS	Reference		Reference	
Intermediate GRS	1.12 (0.98, 1.31)	0.146	1.10 (0.92, 1.30)	0.300
High GRS	1.28 (1.11, 1.50)	0.001	1.32 (1.11, 1.56)	0.002
*P*-value for trend†	0.001		0.002	
Per 10 mm Hg higher	1.00 (0.85, 1.18)	0.989	0.94 (0.78, 1.12)	0.489
**DR**				
Low GRS	Reference		Reference	
Intermediate GRS	1.15 (0.95, 1.38)	0.151	1.17 (0.95, 1.44)	0.143
High GRS	1.43 (1.20, 1.71)	<0.001	1.48 (1.21, 1.82)	<0.001
*P*-value for trend†	<0.001		<0.001	
Per 10 mm Hg higher	0.98 (0.81, 1.19)	0.876	0.94 (0.76, 1.17)	0.574
**DKD**				
Low GRS	Reference		Reference	
Intermediate GRS	1.42 (0.80, 2.54)	0.231	1.35 (0.71, 2.55)	0.357
High GRS	2.51 (1.49, 4.23)	0.001	2.80 (1.57, 4.98)	<0.001
*P*-value for trend†	<0.001		<0.001	
Per 10 mm Hg higher	1.99 (1.18, 3.37)	0.010	2.47 (1.39, 4.40)	0.002
**DN**				
Low GRS	Reference		Reference	
Intermediate GRS	0.98 (0.75, 1.30)	0.914	0.94 (0.70, 1.28)	0.705
High GRS	0.97 (0.74, 1.28)	0.849	1.04 (0.77, 1.42)	0.777
*P*-value for trend†	0.849		0.776	
Per 10 mm Hg higher	1.04 (0.77, 1.39)	0.819	0.90 (0.65, 1.25)	0.537

GRS – genetic risk score, DMCs – diabetic microvascular complications, DR – diabetic retinopathy, DKD – diabetic kidney disease, DN – diabetic neuropathy, BP – blood pressure, HR – hazard ratio, CI – confidence interval

*All models were adjusted for age, sex, body mass index, ethnicity, Townsend index, smoking status, alcohol consumption, glycosylated haemoglobin, duration of diabetes, anti-hyperglycaemic medication, and anti-hypertensive medication. The following additional adjustments to individual models were made: for DMCs, low-density lipoprotein cholesterol and estimated glomerular filtration rate; for DR, low-density lipoprotein cholesterol; for DKD, estimated glomerular filtration rate; and for DN, education level and low-density lipoprotein cholesterol.

†Values with *P* < 0.05 are considered statistically significant.

### Blood pressure classification and diabetic microvascular complications

The characteristics of the participants with diabetes and the DMCs incidence for JNC 7 and 2017 ACC/AHA guidelines for hypertension are presented in **Table 5**. The DMCs incidences significantly differed among diabetes participants of different age group, sex, BMI level, HbA1c level, TC level, and drinking status (all *P* < 0.05). There was no significant difference in DMCs incidence among participants defined by the JNC 7 or the 2017 ACC/AHA guidelines.

**Table 5:** Characteristics of the participants with diabetes and the incidence of microvascular complications by both guidelines for hypertension

Variables	Overall diabetes		JNC 7 defined hypertension		2017 ACC/AHA defined hypertension		Difference, % (95% CI)	*P*-value
	**No**	**Incident (95% CI)**	**No**	**Incident (95% CI)**	**No**	**Incident (95% CI)**		
**Age in years**								
40-44	39	3.45 (2.47, 4.69)	21	5.20 (3.25, 7.84)	33	4.39 (3.04, 6.12)	-0.81 (-3.40, 1.81)	0.537
45-54	197	4.11 (3.57, 4.72)	103	4.71 (3.86, 5.68)	163	4.38 (3.74, 5.09)	-0.33 (-1.43, 0.78)	0.559
55-64	476	4.44 (4.06, 4.85)	278	4.64 (4.12, 5.21)	392	4.44 (3.74, 5.09)	-0.20 (-0.89, 0.48)	0.560
≥65	348	5.44 (4.90, 6.03)	207	5.16 (4.49, 5.89)	293	5.33 (4.75, 5.96)	0.17 (-0.73, 1.08)	0.707
*P*-value*	0.001							
**Sex**								
Female	383	4.15 (3.75, 4.58)	209	4.48 (3.91, 5.12)	318	4.39 (3.93, 4.89)	-0.09 (-0.85, 0.67)	0.817
Male	677	4.91 (4.55, 5.28)	400	5.04 (4.57, 5.55)	563	4.87 (4.48, 5.28)	-0.17 (-0.79, 0.45)	0.586
*P*-value*	0.007							
**Ethnicity**								
White	916	4.61 (4.32, 4.91)	524	4.74 (4.35, 5.15)	763	4.67 (4.35, 5.00)	-0.07 (-0.58, 0.44)	0.782
Others	144	4.58 (3.87, 5.37)	85	5.51 (4.42, 6.77)	118	4.80 (3.99, 5.73)	-0.71 (-2.12, 0.71)	0.323
*P*-value*	0.938							
**BMI in kg/m^2^**								
<25	106	4.09 (3.36, 4.92)	51	4.63 (3.46, 6.04)	80	4.49 (3.58, 5.56)	-0.14 (-1.71, 1.43)	0.862
25-29.9	334	4.20 (3.77, 4.66)	190	4.37 (3.79, 5.03)	277	4.31 (3.83, 4.84)	-0.06 (-0.85, 0.73)	0.880
≥30	601	4.89 (4.52, 5.29)	358	5.07 (4.57, 5.61)	507	4.86 (4.45, 5.29)	-0.21 (-0.87, 0.44)	0.521
*P*-value*	0.033							
**HbA1c in mmol/mol**								
<48	242	2.86 (2.51, 3.24)	130	3.00 (2.51, 3.55)	192	2.90 (2.51, 3.33)	-0.10 (-0.75, 0.55)	0.756
≥48	818	5.62 (5.25, 6.00)	479	5.80 (5.30, 6.32)	689	5.66 (5.26, 6.08)	-0.14 (-0.79, 0.51)	0.679
*P*-value*	<0.001							
**TG in mmol/L**								
<2.3	607	4.40 (4.06, 4.75)	347	4.83 (4.35, 5.35)	505	4.59 (4.21, 5.00)	-0.24 (-0.87, 0.40)	0.461
≥2.3	453	4.91 (4.48, 5.37)	262	4.84 (4.28, 5.45)	376	4.82 (4.35, 5.32)	-0.02 (-0.77, 0.72)	0.949
*P*-value*	0.070							
**TC in mmol/L**								
<5.17	735	4.87 (4.53, 5.23)	413	5.23 (4.75, 5.75)	607	5.02 (4.64, 5.43)	-0.21 (-0.84, 0.42)	0.510
≥5.17	325	4.09 (3.67, 4.55)	196	4.17 (3.62, 4.78)	274	4.08 (3.62, 4.58)	-0.09 (-0.83, 0.66)	0.819
*P*-value*	0.007							
**LDL-C, mmol/L**								
<4.1	892	4.59 (4.30, 4.90)	514	4.94 (4.53, 5.37)	741	4.71 (4.39, 5.06)	-0.23 (-0.76, 0.31)	0.406
≥4.1	168	4.65 (3.99, 5.39)	95	4.35 (3.53, 5.29)	140	4.55 (3.84, 5.35)	0.20 (-0.92, 1.33)	0.724
*P*-value*	0.883							
**HDL-C, mmol/L**								
<1.0	254	4.55 (4.25, 4.87)	143	5.07 (4.29, 5.95)	202	4.69 (4.08, 5.36)	-0.38 (-1.41, 0.64)	0.459
≥1.0	806	4.76 (4.21, 5.37)	466	4.77 (4.35, 5.21)	679	4.69 (4.35, 5.04)	-0.08 (-0.63, 0.46)	0.772
*P*-value*	0.527							
**Smoking status**								
Never	472	4.40 (4.02, 4.80)	267	4.66 (4.13, 5.24)	408	4.69 (4.25, 5.16)	0.03 (-0.68, 0.73)	0.937
Former/current	588	4.78 (4.41, 5.17)	342	4.98 (4.48, 5.52)	473	4.68 (4.28, 5.11)	-0.30 (-0.96, 0.36)	0.376
*P*-value*	0.163							
**Alcohol consumption**								
Never	118	5.65 (4.70, 6.73)	65	6.11 (4.75, 7.72)	95	5.78 (4.70, 7.02)	-0.33 (-2.16, 1.50)	0.722
Former/current	942	4.50 (4.22, 4.79)	544	4.72 (4.34, 5.12)	786	4.58 (4.27, 4.91)	-0.14 (-0.63, 0.36)	0.592
*P*-value*	0.017*							

BMI – body mass index, HbA1c – glycosylated haemoglobin, TG – triglycerides, TC – total cholesterol, LDL-C – low-density lipoprotein cholesterol, HDL-C – high-density lipoprotein cholesterol, CI – confidence interval

*Values with *P* < 0.05 are considered statistically significant.

**Figure 3:**
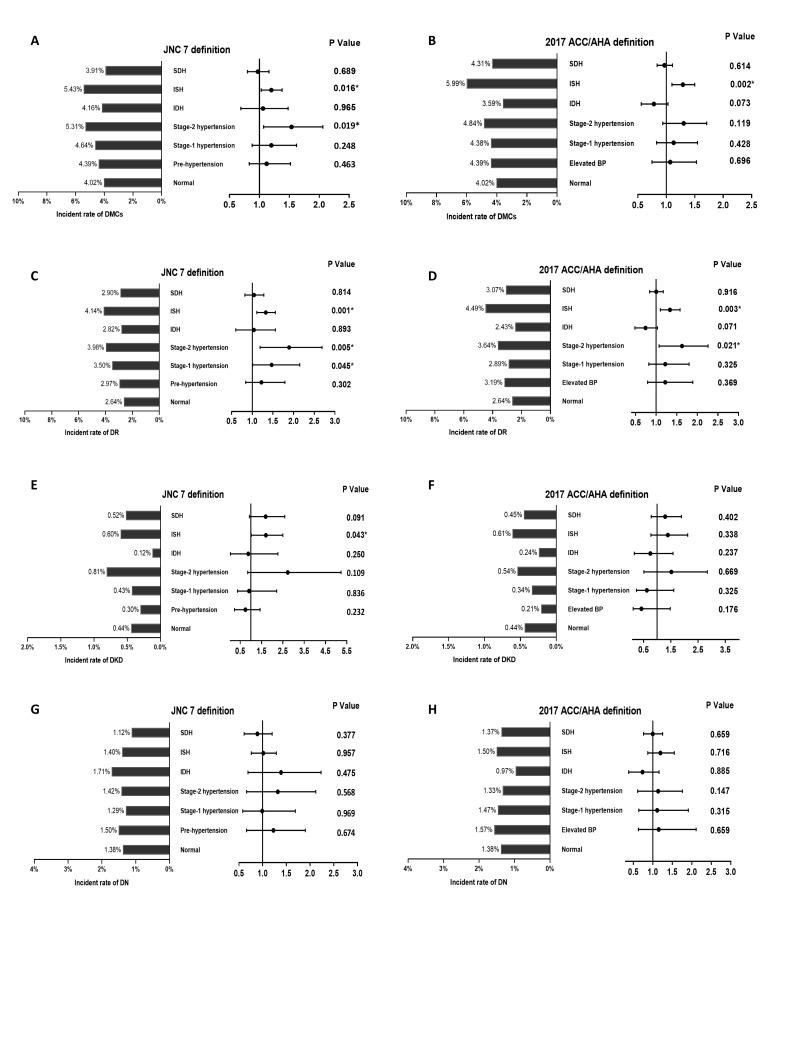
Incidence rate and Cox proportional hazards models for (A-B) DMCs, (C-D) DR, (E, F) DKD, and (G, H) DN among different blood pressure categories defined by JNC7 (A, C, E, G) and 2017 ACC/AHA (B, D, F, H) guidelines. **Panel A. **Incidence rate and Cox proportional hazards models for DMCs among different blood pressure categories defined by JNC7. **Panel B. **Incidence rate and Cox proportional hazards models for DMCs among different blood pressure categories defined by 2017 ACC/AHA. **Panel C. **Incidence rate and Cox proportional hazards models for DR among different blood pressure categories defined by JNC7. **Panel D. **Incidence rate and Cox proportional hazards models for DR among different blood pressure categories defined by 2017 ACC/AHA. **Panel E. **Incidence rate and Cox proportional hazards models for DKD among different blood pressure categories defined by JNC7. **Panel F. **Incidence rate and Cox proportional hazards models for DKD among different blood pressure categories defined by 2017 ACC/AHA. **Panel G. **Incidence rate and Cox proportional hazards models for DN among different blood pressure categories defined by JNC7. **Panel H. **Incidence rate and Cox proportional hazards models for DN among different blood pressure categories defined by 2017 ACC/AHA; Adjusted for age, sex, body mass index, ethnicity, Townsend index, smoking status, alcohol consumption, glycosylated haemoglobin, low-density lipoprotein cholesterol, duration of diabetes, anti-hyperglycaemic medication, anti-hypertensive medication, and estimated glomerular filtration rate. DMCs – diabetic microvascular complications, DR – diabetic retinopathy, DKD – diabetic kidney disease, DN – diabetic neuropathy, IDH – isolated diastolic hypertension, ISH – isolated systolic hypertension, SDH – systolic-diastolic hypertension.

We further explored the association between BP classification defined by the JNC7 and 2017 ACC/AHA guidelines and incidence of DMCs, DR, DKD, and DN using Cox proportional hazards models (Tables S6-S9 in the **Online Supplementary Document** and **Figure 3**, panels A-H). Participants with higher BP classification had a higher risk of incidence of DMCs and DR, compared with normal stage. With the increase in BP classification, the risk trends for incidence of DR and DKD significantly increased, but not for incidence of DN. For the different categories of hypertension, participants with ISH had higher risk of incidence of DMCs, DR, and DKD.

Additionally, we conducted subgroup analyses stratified by sex, HbA1c level, and use of anti-hyperglycaemic and anti-hypertensive medication to examine the relationship between BP and DMCs incidence using multivariable Cox models (Figure S1-S5 in the **Online Supplementary Document**). In the female subgroup, participants with SBP 130-139, 150-159, and ≥160 mm Hg had higher risk of DMCs, compared to SBP<120 mm Hg. In the subgroup of HbA1c ≥48 mmol/mol, participants with SBP 150-159 and ≥160 mm Hg had higher risk of DMCs. No significant difference was observed for subgroup of HbA1c <48 mmol/mol. For the subgroup of using medication, the association between SBP and risk of DMCs incidence was only found in the participants with anti-hyperglycaemic or anti-hypertensive medication separately or combine.

### Sensitivity analysis

To explore the cumulative effect of different types of DMCs, we compared the risk of incidence of single and multiple DMCs with the increase in BP (Figure S6 in the **Online Supplementary Document**). The associations of SBP were stronger for those with two or more DMCs compared with those with only one DMCs. Considering that insulin therapy was an independent risk factor for diabetic complications [23], and patients with insulin use are usually sicker and develop complications earlier, we examined the association of different BP levels and BP stage by adjusting for use of insulin rather than of anti-hyperglycaemic medication (Table S10 in the **Online Supplementary Document**). However, the results were similar to those gained after adjusting for anti-hyperglycaemic medication. Additionally, a similar pathological basis existed in DKD and DR, which differed from DN [24]. Therefore, we further tested the association between BP and DMCs incidence after excluding patients with DN (Tables S11 and S12 in the **Online Supplementary Document**). The exclusion had little influence on the above associations.

## DISCUSSION

In this large cohort study, higher levels of SBP were significantly associated with an increased risk of DMCs among patients with diabetes in a dose-response relationship. Moreover, there was no difference in DMCs incidence using either the newly established 2017 AHA/ACC or the JNC 7 guidelines.

Although a relationship between lowering BP and BP variability and the risk of DN has been reported among diabetic populations in several prospective studies [3-5,7,10], retinopathic and neuropathic evidence is either inconsistent or limited [7,8]. In our study, a higher level of SBP was significantly associated with incidence of DMCs, DR, and DKD. Notably, the relationship between BP and DR incidence is controversial. Some studies revealed that higher BP was associated with development of DR [8,25-28], while others found no significant association between BP and DR [9,29-31]. Some possible mechanisms have been proposed by which hypertension could affect DR via haemodynamic changes and vascular endothelial growth factor-dependent pathways [32]. To minimise the influence of confounders, we constructed the GRS for BP, and these results further supported the significant relationship between SBP and DMCs and DR. The dose-response analysis showed nonlinear effects of BP on incidence of DMCs. Furthermore, the more intensive BP control in patients with hypertension and diabetes has benefits in prevention and treatment of DMCs [33]. Therefore, exploring the BP-controlling targets can help in increasing benefits and reducing microvascular damage from diabetes.

Additionally, the effectiveness of glycaemic control and BP management to decrease DMCs remains controversial. The UKPDS showed that, after adjusting confounders, intensive BP control could significantly reduce the risk of DMCs [13]. In contrast, the Appropriate Blood Pressure Control in Diabetes Trial demonstrated no difference in DMCs progression over five years between the intensive and moderate BP control groups [33]. A systematic review of 15 randomised trials supported the hypothesis that BP control could reduce the incidence, but not the progression of DR [34]. Similarly, some studies have suggested that intensive glycaemic control reduced DN incidence [35,36], while others found no significant decrease in DN incidence after intensive glycaemic control for type 2 diabetes mellitus [37]. Our subgroup analyses showed that the association between BP and DMCs was more evident in patients using anti-hyperglycaemic or anti-hypertensive medication or a combination, which might result from the severity of the underlying disease rather than the consequence of treatment status. Further studies should explore the efficacy of BP and glycaemic control and the extent of reduction needed for beneficial effects. Moreover, we found that the relationship between SBP and DMCs was significant only in females. Previous studies suggested that the risk for vascular complications appear to be greater for diabetic females than males; the genetic, sex hormones, and sex-specific risk factors might explain the sex differences of DMCs [38,39]. Nonetheless, the reasons for this different impact between sex remain unclear and deserve further investigation.

According to the 2017 AHA/ACC guideline, lowering the cut-off to define hypertension may increase the number of populations with hypertension, and is estimated to classify approximately 46% of the US adult population as having hypertension [40]. However, whether the stricter definition will affect the incidence of DMCs in relation to hypertension remains unclear. Our results suggested that the new guideline on hypertension may not affect the incidence of DMCs in the UK population with diabetes. Future studies on other racial populations are needed to verify this impact.

Our study showed that diabetic patients with higher SBP levels had a higher risk of DMCs. This transition requires clinical and preventative strategies for BP control among patients with diabetes. If confirmed by replication, our findings may have implications for DMCs prevention strategies that target improving and maintaining BP measurements among patients with diabetes. They also contribute to the scientific basis for the development of intervention studies for future DMCs prevention among patients with diabetes.

A major strength of our study is that the UK Biobank study is a prospective long-term cohort study which has collected extensive phenotypic and genotypic data. Therefore, we made meticulous adjustments for a wide range of potential confounding factors. However, our study also has several potential limitations. First, the ICD code may be insufficient for detecting cases in an early stage or to classifying DR, DKD, and DN case subtypes. Second, we used the baseline measurement of BP levels in the UK Biobank, so we did not capture changes in BP levels during follow-up, which may lead to non-differential misclassification bias. Third, self-reported lifestyle factors and medical history data were subject to measurement error, which may lead to misclassification bias. Fourth, our study was limited to the UK population with diabetes who were aged 40-69 years, thus, our findings may not be directly generalisable to other populations. Further studies with a wider age range are needed. Additionally, the sample sizes for DKD and DN were relatively small, which might have limited power for non-significant associations. Finally, six different hypertension management guidelines have been published by American (JNC7, 2017 ACC/AHA), European (2013/2018 ESH/ESC), and international organisations (WHO/International Society of Hypertension (ISH) 2003, ISH 2020); we only compared the DMCs differences between JNC7 and 2017 AHA/ACC, because the 2017 AHA/ACC is the only one that does not define hypertension as 140/90 mm Hg.

## CONCLUSIONS

In this long-term follow-up, large-scale, prospective cohort study of a population with diabetes, both genetic and epidemiological evidence suggested that higher levels of SBP were significantly associated with an increased risk of DMCs. Furthermore, high BP, defined as SBP/DBP of at least 130/80 mm Hg using the 2017 AHA/ACC guidelines, did not influence the incidence of DMCs. The findings suggest that BP control among patients with diabetes may be useful in DMCs care and prevention.

## Additional material


Online Supplementary Document
