# Supplementary material for: Association of blood pressure with incident diabetic microvascular complications among diabetic patients: Longitudinal findings from the UK Biobank
Source: J Glob Health. 2023 Mar 24;13:04027. doi: 10.7189/jogh.13.04027 (PMC10039372; doi:10.7189/jogh.13.04027)
Supplement: Online Supplementary Document [file jogh-13-04027-s001.pdf]

Table S1. Blood pressure classifications by JNC 7 and 2017 ACC/AHA guidelines

Systolic BP (mmHg)	Diastolic BP (mmHg)	JCN7	2017 ACC/AHA
<120	And <80	Normal	Normal
120–129	And <80	Pre-hypertension	Elevated BP
130–139	Or 80–89	Pre-hypertension	Stage 1-hypertension
140–159	Or 90–99	Stage 1-hypertension	Stage 2-hypertension
≥160	Or ≥100	Stage 2-hypertension	Stage 2-hypertension
<140	And ≥90	IDH	
≥140	And <90	ISH	
≥140	And ≥90	SDH	
<130	And ≥80		IDH
≥130	And <80		ISH
≥130	And ≥80		SDH

JNC7, Seventh Report of the Joint National Committee; ACC/AHA, American College of Cardiology/American Heart Association; BP, blood pressure; IDH, isolated diastolic hypertension; ISH, isolated systolic hypertension; SDH, systolic–diastolic hypertension.

Table S2. Disease codes for diabetic microvascular complications in UK Biobank

Disease	Diagnoses of ICD-10	Diagnoses of ICD-9	Non-cancer illness
Diabetic retinopathy	H360, H280, E133, E143, E103, E113, E123	3620,2504	1276
Diabetic kidney disease	E112, E102, E122, E132, E142	2503	1607
Diabetic neuropathy	E104, E114, E124, E134, E144, G590, G632, G990	2505, 3572	1468

ICD, International Classification of Diseases.

Table S3. Cox proportional hazards models for incident diabetic retinopathy by different blood pressure levels

**Blood pressure level (mmHg)**	**Crude HR (95% CI)**	***P***	**Adjusted HR (95% CI)**	***P***
**Systolic blood pressure**				
<120	reference		reference	
120–129	1.09 (0.79-1.51)	0.605	1.20 (0.80-1.78)	0.381
130–139	1.13 (0.83-1.54)	0.443	1.34 (0.92-1.96)	0.128
140–149	1.20 (0.88-1.64)	0.242	1.44 (0.99-2.12)	0.060
150–159	1.53 (1.12-2.10)	0.008*	1.73 (1.17-2.56)	0.006*
≥160	1.55 (1.13-2.13)	0.007*	1.84 (1.23-2.73)	0.003*
P for trend	<0.001*		<0.001*	
Per 10 mmHg higher at baseline	1.10 (1.05-1.14)	<0.001*	1.12 (1.06-1.17)	<0.001*
**Diastolic blood pressure**				
<80	reference		reference	
80–89	0.80 (0.68-0.94)	0.005*	0.89 (0.75-1.08)	0.247
90–99	0.70 (0.56-0.86)	0.001*	0.85 (0.66-1.09)	0.189
≥100	0.99 (0.70-1.42)	0.977	1.60 (1.09-2.35)	0.015*
P for trend	0.006*		0.913	
Per 10 mmHg higher at baseline	0.87 (0.81-0.94)	0.001*	0.99 (0.90-1.08)	0.802

Adjusted for age, sex, body mass index, ethnicity, Townsend index, smoking status, alcohol consumption, glycosylated haemoglobin, low-density lipoprotein cholesterol, duration of diabetes, anti-hyperglycaemic medication, and anti-hypertensive medication.

* Refers to statistically significant.

Table S4. Cox proportional hazards models for incident diabetic kidney disease by different blood pressure levels

Blood pressure level (mmHg)	Crude HR (95% CI)	*P*	Adjusted HR (95% CI)	*P*
**Systolic blood pressure**				
<120	reference		reference	
120–129	0.47 (0.18-1.22)	0.121	0.40 (0.13-1.20)	0.103
130–139	0.75 (0.34-1.67)	0.482	0.61 (0.24-1.55)	0.304
140–149	0.89 (0.41-1.96)	0.779	0.89 (0.36-2.18)	0.802
150–159	1.22 (0.55-2.71)	0.617	1.08 (0.43-2.73)	0.868
≥160	1.97 (0.93-4.17)	0.077	2.28 (0.96-5.41)	0.062
P for trend	<0.001*		<0.001*	
Per 10 mmHg higher at baseline	1.24 (1.12-1.37)	<0.001*	1.31 (1.17-1.47)	<0.001*
**Diastolic blood pressure**				
<80	reference		reference	
80–89	0.86 (0.55-1.34)	0.512	0.95 (0.57-1.58)	0.846
90–99	0.85 (0.48-1.49)	0.567	1.09 (0.58-2.04)	0.792
≥100	1.42 (0.60-3.33)	0.425	2.22 (0.91-5.41)	0.081
P for trend	0.959		0.283	
Per 10 mmHg higher at baseline	0.99 (0.80-1.21)	0.892	1.13 (0.89-1.44)	0.332

Adjusted for age, sex, body mass index, ethnicity, Townsend index, smoking status, alcohol consumption, glycosylated haemoglobin, duration of diabetes, anti-hyperglycaemic medication, anti-hypertensive medication, and estimated glomerular filtration rate.

HR, hazard ratio; CI, confidence interval.

* Refers to statistically significant.

Table S5. Cox proportional hazards models for incident diabetic neuropathy by different blood pressure levels

**Blood pressure level (mmHg)**	**Crude HR (95% CI)**	***P***	**Adjusted HR (95% CI)**	***P***
**Systolic blood pressure**				
<120	reference		reference	
120–129	0.97 (0.61-1.53)	0.882	0.93 (0.54-1.62)	0.808
130–139	1.20 (0.78-1.83)	0.409	1.22 (0.73-2.03)	0.444
140–149	0.85 (0.54-1.33)	0.469	0.84 (0.49-1.44)	0.530
150-159	0.99 (0.62-1.58)	0.980	1.09 (0.63-1.89)	0.760
≥160	1.04 (0.65-1.66)	0.883	1.14 (0.65-2.00)	0.637
P for trend	0.804		0.697	
Per 10 mmHg higher at baseline	1.00 (0.93-1.06)	0.916	1.01 (0.95-1.10)	0.607
**Diastolic blood pressure**				
<80	reference		reference	
80–89	0.92 (0.72-1.17)	0.493	0.89 (0.67-1.17)	0.394
90–99	0.78 (0.57-1.08)	0.140	0.82 (0.57-1.18)	0.284
≥100	0.89 (0.49-1.60)	0.691	1.11 (0.60-2.05)	0.732
P for trend	0.180		0.502	
Per 10 mmHg higher at baseline	0.93 (0.83-1.05)	0.251	0.98 (0.85-1.12)	0.748

Adjusted for age, sex, body mass index, ethnicity, Townsend index, smoking status, alcohol consumption, education level, glycosylated haemoglobin, low-density lipoprotein cholesterol, duration of diabetes, anti-hyperglycaemic medication and anti-hypertensive medication.

HR, hazard ratio; CI, confidence interval.

* Refers to statistically significant.

Table S6. Cox proportional hazards models for incident diabetic microvascular complications by both guidelines for blood pressure classification

Blood pressure classification	Crude HR (95% CI)	*P*	Adjusted HR (95% CI)	*P*
**JNC 7**				
Normal	reference		reference	
Pre-hypertension	1.08 (0.84-1.39)	0.550	1.12 (0.83-1.52)	0.463
Stage-1 hypertension	1.14 (0.89-1.47)	0.289	1.20 (0.88-1.62)	0.248
Stage-2 hypertension	1.32 (1.01-1.73)	0.043*	1.48 (1.07-2.06)	0.019*
P for trend	0.015*		0.004*	
IDH	0.88 (0.63-1.24)	0.478	1.01 (0.69-1.48)	0.965
ISH	1.31 (1.16-1.48)	<0.001*	1.19 (1.03-1.38)	0.016*
SDH	0.81 (0.69-0.96)	0.013 *	0.96 (0.80-1.16)	0.689
**2017 ACC/AHA**				
Normal	reference		reference	
Elevated blood	1.09 (0.81-1.47)	0.580	1.07 (0.75-1.53)	0.696
Stage-1 hypertension	1.08 (0.83-1.39)	0.577	1.13 (0.83-1.55)	0.428
Stage-2 hypertension	1.19 (0.94-1.52)	0.152	1.27 (0.94-1.71)	0.119
P for trend	0.073		0.035*	
IDH	0.75 (0.58-0.98)	0.035*	0.76 (0.56-1.03)	0.073
ISH	1.45 (1.26-1.65)	<0.001*	1.28 (1.10-1.50)	0.002*
SDH	0.86 (0.76-0.97)	0.015*	0.96 (0.84-1.11)	0.614

Adjusted for age, sex, body mass index, ethnicity, Townsend index, smoking status, alcohol consumption, glycosylated haemoglobin, low-density lipoprotein cholesterol, duration of diabetes, anti-hyperglycaemic medication, anti-hypertensive medication, and estimated glomerular filtration rate.

IDH, isolated diastolic hypertension; ISH, isolated systolic hypertension.

IDH, isolated diastolic hypertension; ISH, isolated systolic hypertension; SDH, systolic–diastolic hypertension; HR, hazard ratio; CI, confidence interval.

* Refers to statistically significant.

Table S7. Cox proportional hazards models for incident diabetic retinopathy by both guidelines for blood pressure classification

Blood pressure classification	Crude HR (95% CI)	*P*	Adjusted HR (95% CI)	*P*
**JNC 7**				
Normal	reference		reference	
Pre-hypertension	1.11 (0.82-1.51)	0.509	1.22 (0.84-1.79)	0.302
Stage-1 hypertension	1.31 (0.97-1.78)	0.080	1.47 (1.01-2.15)	0.045*
Stage-2 hypertension	1.50 (1.08-2.08)	0.015*	1.79 (1.19-2.69)	0.005*
P for trend	0.001*		0.001*	
IDH	0.83 (0.55-1.26)	0.382	0.96 (0.60-1.56)	0.893
ISH	1.45 (1.25-1.67)	<0.001*	1.31 (1.11-1.56)	0.001*
SDH	0.85 (0.70-1.02)	0.087	1.03 (0.82-1.28)	0.814
**2017 ACC/AHA**				
Normal	reference		reference	
Elevated blood	1.20 (0.84-1.72)	0.320	1.22 (0.79-1.89)	0.369
Stage-1 hypertension	1.07 (0.78-1.48)	0.659	1.22 (0.82-1.80)	0.325
Stage-2 hypertension	1.37 (1.01-1.84)	0.040*	1.55 (1.07-2.26)	0.021*
P for trend	0.009*		0.002*	
IDH	0.71 (0.52-0.98)	0.035*	0.70 (0.48-1.03)	0.071
ISH	1.53 (1.31-1.79)	<0.001*	1.32 (1.10-1.58)	0.003*
SDH	0.85 (0.74-0.98)	0.024*	0.99 (0.84-1.17)	0.916

Adjusted for age, sex, ethnicity, Townsend index, smoking status, alcohol consumption, glycosylated haemoglobin, low-density lipoprotein cholesterol, duration of diabetes, anti-hyperglycaemic medication, and anti-hypertension medication.

IDH, isolated diastolic hypertension; ISH, isolated systolic hypertension; SDH, systolic–diastolic hypertension; HR, hazard ratio; CI, confidence interval.

* Refers to statistically significant.

Table S8. Cox proportional hazards models for incident diabetic kidney disease by both guidelines for blood pressure classification

Blood pressure classification	Crude HR (95% CI)	*P*	Adjusted HR (95% CI)	*P*
**JNC 7**				
Normal	reference		reference	
Pre-hypertension	0.67 (0.31-1.49)	0.330	0.57 (0.23-1.43)	0.232
Stage-1 hypertension	0.97 (0.45-2.07)	0.936	0.91 (0.37-2.21)	0.836
Stage-2 hypertension	1.82 (0.83-3.99)	0.133	2.10 (0.85-5.23)	0.109
P for trend	0.003*		<0.001*	
IDH	0.26 (0.04-1.89)	0.185	0.31 (0.04-2.27)	0.250
ISH	1.65 (1.11-2.43)	0.012*	1.59 (1.02-2.50)	0.043*
SDH	1.20 (0.75-1.92)	0.450	1.55 (0.93-2.59)	0.091
**2017 ACC/AHA**				
Normal	reference		reference	
Elevated blood	0.47 (0.15-1.42)	0.180	0.42 (0.12-1.48)	0.176
Stage-1 hypertension	0.75 (0.33-1.70)	0.498	0.62 (0.24-1.61)	0.325
Stage-2 hypertension	1.21 (0.58-2.52)	0.608	1.21 (0.51-2.84)	0.669
P for trend	0.062		0.047*	
IDH	0.52 (0.19-1.43)	0.206	0.50 (0.16-1.58)	0.237
ISH	1.55 (1.01-2.37)	0.044*	1.28 (0.77-2.12)	0.338
SDH	1.02 (0.69-1.51)	0.906	1.21 (0.78-1.89)	0.402

Adjusted for age, sex, body mass index, ethnicity, Townsend index, smoking status, alcohol consumption, glycosylated haemoglobin, duration of diabetes, anti-hyperglycaemic medication, anti-hypertensive medication, and estimated glomerular filtration rate.

IDH, isolated diastolic hypertension; ISH, isolated systolic hypertension; SDH, systolic–diastolic hypertension; HR, hazard ratio; CI, confidence interval.

* Refers to statistically significant.

Table S9. Cox proportional hazards models for incident diabetic neuropathy by both guidelines for blood pressure classification

**Blood pressure classification**	**Crude HR (95% CI)**	***P***	**Adjusted HR (95% CI)**	***P***
**JNC 7**				
Normal	reference		reference	
Pre-hypertension	1.07 (0.70-1.65)	0.747	1.12 (0.66-1.90)	0.674
Stage-1 hypertension	0.92 (0.60-1.42)	0.718	0.99 (0.58-1.69)	0.969
Stage-2 hypertension	1.02 (0.63-1.65)	0.926	1.18 (0.66-2.12)	0.568
P for trend	0.616		0.890	
IDH	1.22 (0.71-2.09)	0.465	1.24 (0.69-2.23)	0.475
ISH	1.01 (0.80-1.28)	0.920	0.99 (0.76-1.29)	0.957
SDH	0.77 (0.56-1.04)	0.088	0.86 (0.61-1.20)	0.377
**2017 ACC/AHA**				
Normal	reference		reference	
Elevated blood	1.14 (0.69-1.88)	0.621	1.15 (0.63-2.11)	0.659
Stage-1 hypertension	1.05 (0.67-1.63)	0.833	1.11 (0.64-1.91)	0.716
Stage-2 hypertension	0.95 (0.63-1.45)	0.817	1.04 (0.61-1.76)	0.885
P for trend	0.436		0.806	
IDH	0.67 (0.41-1.11)	0.124	0.66 (0.38-1.16)	0.147
ISH	1.11 (0.86-1.44)	0.425	1.16 (0.87-1.55)	0.315
SDH	0.96 (0.77-1.20)	0.723	0.98 (0.76-1.25)	0.846

Adjusted for age, sex, body mass index, ethnicity, Townsend index, smoking status, alcohol consumption, education level, glycosylated haemoglobin, low-density lipoprotein cholesterol, duration of diabetes, anti-hyperglycaemic medication, and anti-hypertensive medication.

IDH, isolated diastolic hypertension; ISH, isolated systolic hypertension; SDH, systolic–diastolic hypertension; HR, hazard ratio; CI, confidence interval.

* Refers to statistically significant.

Table S10. Cox proportional hazards models for incident diabetic microvascular complications by different blood pressure levels and blood pressure classification by both guidelines, especially adjusted for insulin use

**Blood pressure level (mmHg)**	**Adjusted HR (95% CI)**	***P***
**Systolic blood pressure**		
<120	reference	
120–129	1.02 (0.74-1.40)	0.912
130–139	1.20 (0.89-1.62)	0.223
140–149	1.11 (0.82-1.51)	0.504
150–159	1.30 (0.95-1.78)	0.103
≥160	1.44 (1.05-1.98)	0.024*
P for trend	0.003*	
Per 10 mmHg higher at baseline	1.08 (1.03-1.12)	<0.001*
**Diastolic blood pressure**		
<80	reference	
80–89	0.89 (0.76-1.04)	0.134
90–99	0.81 (0.66-1.00)	0.054
≥100	1.27 (0.91-1.78)	0.160
P for trend	0.410	
Per 10 mmHg higher at baseline	0.96 (0.89-1.04)	0.285
**Blood pressure classification**		
**JNC 7**		
Normal	reference	
Pre-hypertension	1.13 (0.83-1.53)	0.434
Stage-1 hypertension	1.19 (0.88-1.61)	0.269
Stage-2 hypertension	1.44 (1.03-2.00)	0.031*
P for trend	0.012*	
IDH	1.00 (0.69-1.47)	0.982
ISH	1.18 (1.03-1.37)	0.021*
SDH	0.94 (0.78-1.13)	0.502
**2017 ACC/AHA**		
Normal	reference	
Elevated blood	1.08 (0.76-1.54)	0.660
Stage-1 hypertension	1.14 (0.84-1.56)	0.401
Stage-2 hypertension	1.25 (0.93-1.69)	0.144
P for trend	0.060	
IDH	0.77 (0.57-1.05)	0.104
ISH	1.29 (1.11-1.51)	0.001*
SDH	0.95 (0.82-1.09)	0.432

Adjusted for age, sex, body mass index, ethnicity, Townsend index, smoking status, alcohol consumption, glycosylated haemoglobin, low-density lipoprotein cholesterol, duration of diabetes, insulin use, anti-hypertensive medication, and estimated glomerular filtration rate.

IDH, isolated diastolic hypertension; ISH, isolated systolic hypertension; SDH, systolic–diastolic hypertension; HR, hazard ratio; CI, confidence interval.

* Refers to statistically significant.

Table S11. Cox proportional hazards models for incident diabetic microvascular complications by different blood pressure levels after excluding DN

**Blood pressure level (mmHg)**	**Crude HR (95% CI)**	***P***	**Adjusted HR (95% CI)**	***P***
**Systolic blood pressure**				
<120	reference		reference	
120–129	1.03 (0.74-1.43)	0.862	1.02 (0.69-1.51)	0.913
130–139	1.12 (0.83-1.52)	0.463	1.16 (0.80-1.68)	0.433
140–149	1.18 (0.87-1.61)	0.285	1.18 (0.81-1.71)	0.379
150–159	1.44 (1.05-1.97)	0.024*	1.38 (0.94-2.03)	0.101
≥160	1.55 (1.13-2.12)	0.006*	1.62 (1.10-2.39)	0.014*
P for trend	<0.001*		0.001*	
Per 10 mmHg higher at baseline	1.10 (1.05-1.14)	<0.001*	1.10 (1.05-1.16)	<0.001*
**Diastolic blood pressure**				
<80	reference		reference	
80–89	0.79 (0.67-0.93)	0.005*	0.91 (0.76-1.10)	0.164
90–99	0.66 (0.53-0.82)	<0.001*	0.82 (0.64-1.06)	0.089
≥100	0.89 (0.62-1.29)	0.554	1.39 (0.93-2.08)	0.085
P for trend	0.001*		0.706	
Per 10 mmHg higher at baseline	0.85 (0.78-0.92)	<0.001*	0.96 (0.87-1.05)	0.343

Adjusted for age, sex, body mass index, ethnicity, Townsend index, smoking status, alcohol consumption, glycosylated haemoglobin, low-density lipoprotein cholesterol, duration of diabetes, anti-hyperglycaemic medication, anti-hypertensive medication, and estimated glomerular filtration rate.
DN, diabetic neuropathy; HR, hazard ratio; CI, confidence interval.
* Refers to statistically significant.

Table S12. Cox proportional hazards models for incident diabetic microvascular complications by both guidelines for hypertension after excluding DN

**Blood pressure classification**	**Crude HR (95% CI)**	***P***	**Adjusted HR (95% CI)**	***P***
**JNC 7**				
Normal	reference		reference	
Pre-hypertension	1.08 (0.75-1.57)	0.676	1.12 (0.83-1.52)	0.463
Stage-1 hypertension	1.24 (0.85-1.79)	0.263	1.20 (0.88-1.62)	0.248
Stage-2 hypertension	1.57 (1.06-2.34)	0.026*	1.48 (1.07-2.06)	0.019*
P for trend	0.002*		0.004*	
IDH	0.73 (0.47-1.13)	0.161	0.96 (0.58-1.58)	0.866
ISH	1.48 (1.28-1.72)	<0.001*	1.29 (1.09-1.53)	0.004*
SDH	0.81 (0.66-0.98)	0.032*	0.96 (0.77-1.20)	0.730
**2017 ACC/AHA**				
Normal	reference		reference	
Elevated blood	1.03 (0.67-1.59)	0.892	1.07 (0.75-1.53)	0.696
Stage-1 hypertension	1.10 (0.75-1.61)	0.632	1.13 (0.83-1.55)	0.428
Stage-2 hypertension	1.32 (0.92-1.90)	0.136	1.27 (0.94-1.71)	0.119
P for trend	0.020*		0.035*	
IDH	0.75 (0.55-1.02)	0.064	0.79 (0.55-1.14)	0.207
ISH	1.63 (1.39-1.90)	<0.001*	1.29 (1.07-1.56)	0.007*
SDH	0.82 (0.71-0.95)	0.007*	0.96 (0.82-1.14)	0.673

Adjusted for age, sex, body mass index, ethnicity, Townsend index, smoking status, alcohol consumption, glycosylated haemoglobin, low-density lipoprotein cholesterol, duration of diabetes, anti-hyperglycaemic medication, anti-hypertensive medication, and estimated glomerular filtration rate.

IDH, isolated diastolic hypertension; ISH, isolated systolic hypertension; SDH, systolic–diastolic hypertension; DN, diabetic neuropathy; HR, hazard ratio; CI, confidence interval.

* Refers to statistically significant.

Subgroup	No. of patients	Microvascular complication (%)	HR(95%CI)	P-value
**Female**				
**SBP**				
< 120	1099	33 (3.00)	reference	
120–129	1676	58 (3.46)	1.48 (0.84-2.60)	0.173
130–139	2114	97 (4.59)	1.97 (1.16-3.36)	0.012*
140–149	1874	71 (3.79)	1.62 (0.93-2.82)	0.089
150–159	1260	61 (4.84)	1.85 (1.04-3.29)	0.036*
≥ 160	1207	63 (5.22)	2.37 (1.34-4.19)	0.003*
**DBP**				
< 80	4026	181 (4.50)	reference	
80-89	3375	130 (3.85)	0.89 (0.68-1.16)	0.390
90-99	1505	56 (3.72)	0.99 (0.69-1.41)	0.939
≥ 100	324	16 (4.94)	1.54 (0.87-2.73)	0.137
**Male**				
**SBP**				
< 120	949	50 (5.27)	reference	
120–129	2153	97 (4.51)	0.78 (0.53-1.15)	0.216
130–139	3261	150 (4.60)	0.88 (0.61-1.26)	0.471
140–149	3150	144 (4.57)	0.87 (0.61-1.26)	0.469
150–159	2257	121 (5.36)	1.04 (0.72-1.51)	0.836
≥ 160	2030	115 (5.67)	1.16 (0.79-1.69)	0.454
**DBP**				
< 80	5131	293 (5.71)	reference	
80-89	5412	255 (4.71)	0.90 (0.75-1.09)	0.299
90-99	2678	102 (3.80)	0.76 (0.59-0.99)	0.038*
≥ 100	570	27 (4.74)	1.22 (0.81-1.85)	0.343

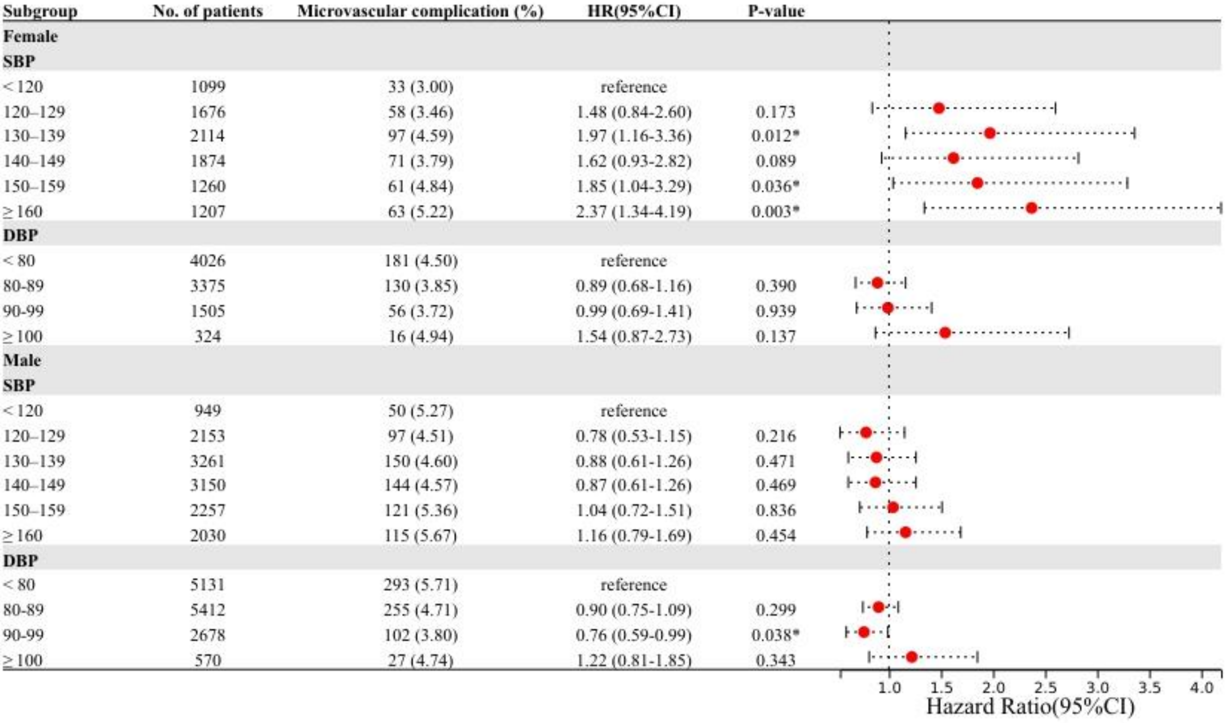

Figure S1. Blood pressure level and incident diabetic microvascular complications stratified by sex

Cox regression models were used to estimate the hazard ratio (95% CI) for incident diabetic microvascular complications associated with blood pressure adjusted for age, body mass index, ethnicity, Townsend index, smoking status, alcohol consumption, glycosylated haemoglobin, low-density lipoprotein cholesterol, duration of diabetes, anti-hyperglycaemic medication, anti-hypertensive medication, and estimated glomerular filtration rate.

SBP, systolic blood pressure; DBP, diastolic blood pressure; HR, hazard ratio; CI, confidence interval.

* Refers to statistically significant.

Subgroup	No. of patients	Microvascular complication (%)	HR (95%CI)	P-value
**HbA1c < 48 mmol/mol**				
**SBP**				
< 120	970	24 (2.47)	reference	
120–129	1493	37 (2.48)	0.84 (0.49-1.46)	0.544
130–139	1931	60 (3.11)	0.93 (0.55-1.56)	0.778
140–149	1761	55 (3.12)	0.99 (0.59-1.66)	0.961
150–159	1233	32 (2.60)	0.81 (0.45-1.43)	0.462
≥ 160	1076	34 (3.16)	1.02 (0.58-1.80)	0.941
**DBP**				
< 80	3700	120 (3.24)	reference	
80-89	3168	78 (2.46)	0.87 (0.58-1.29)	0.477
90-99	1330	35 (2.63)	0.99 (0.60-1.29)	0.985
≥ 100	266	9 (3.38)	1.98 (0.93-4.23)	0.077
**HbA1c ≥ 48 mmol/mol**				
**SBP**				
< 120	1078	59 (5.47)	reference	
120–129	2336	118 (5.05)	1.09 (0.76-1.57)	0.634
130–139	3444	187 (5.43)	1.21 (0.85-1.70)	0.288
140–149	3263	160 (4.90)	1.08 (0.76-1.54)	0.682
150–159	2284	150 (6.57)	1.52 (1.06-2.18)	0.022*
≥ 160	2161	144 (6.66)	1.66 (1.16-2.40)	0.006*
**DBP**				
< 80	5457	354 (6.49)	reference	
80-89	5619	307 (5.46)	0.97 (0.81-1.14)	0.683
90-99	2862	123 (4.30)	0.89 (0.71-1.12)	0.329
≥ 100	628	34 (5.41)	1.35 (0.93-1.98)	0.117

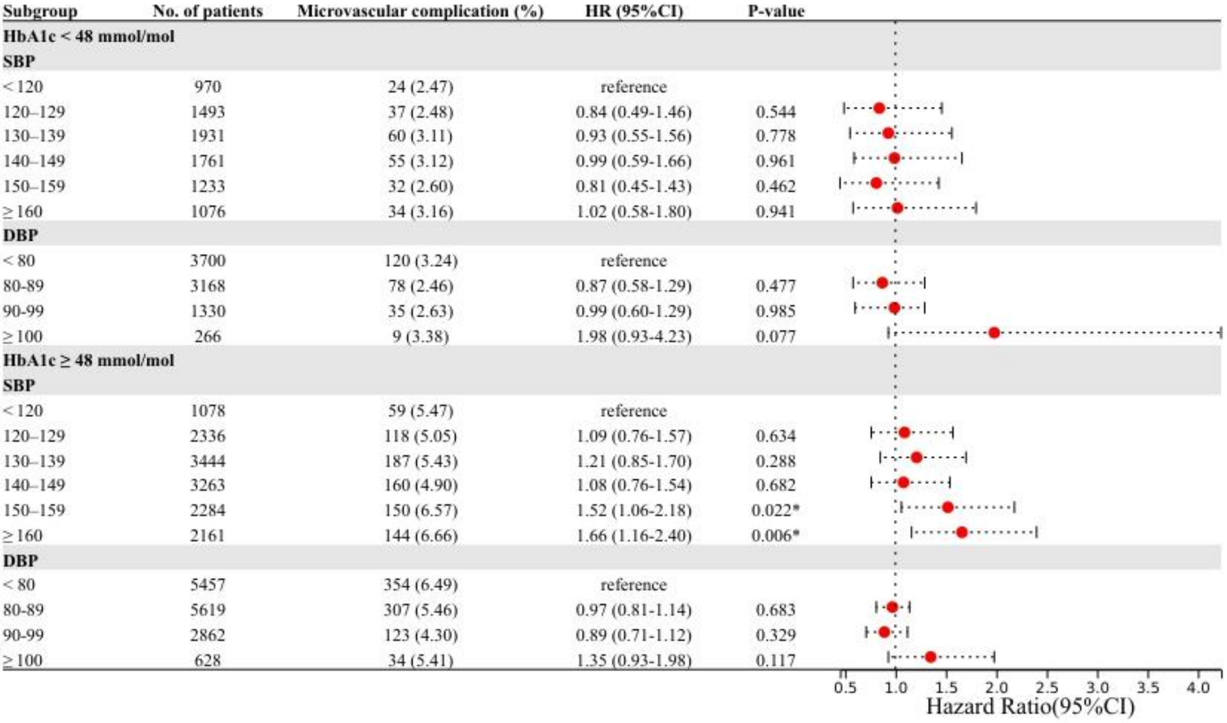

Figure S2. Blood pressure level and incident diabetic microvascular complications stratified by glycosylated haemoglobin

Cox regression models were used to estimate the hazard ratio (95% CI) for incident diabetic microvascular complications associated with blood pressure adjusted for age, sex, body mass index, ethnicity, Townsend index, smoking status, alcohol consumption, low-density lipoprotein cholesterol, duration of diabetes, anti-hyperglycaemic medication, anti-hypertensive medication, and estimated glomerular filtration rate.

SBP, systolic blood pressure; DBP, diastolic blood pressure; HbA1c, glycosylated haemoglobin; HR, hazard ratio; CI, confidence interval.

* Refers to statistically significant.

Subgroup	No. of patients	Microvascular complication (%)	HR (95%CI)	P-value
**Without DM medication**				
**SBP**				
< 120	799	14 (1.75)	reference	
120–129	1493	25 (1.67)	1.04 (0.48-2.26)	0.923
130–139	2155	46 (2.13)	1.13 (0.54-2.36)	0.746
140–149	2092	37 (1.77)	0.85 (0.40-1.82)	0.677
150–159	1617	31 (1.92)	0.80 (0.36-1.78)	0.580
≥ 160	1568	35 (2.23)	0.91 (0.41-2.02)	0.822
**DBP**				
< 80	3303	72 (2.18)	reference	
80-89	3795	65 (1.71)	0.73 (0.49-1.09)	0.121
90-99	2107	38 (1.80)	0.75 (0.47-1.21)	0.245
≥ 100	519	13 (2.50)	1.23 (0.63-2.42)	0.547
**With DM medication**				
**SBP**				
< 120	1249	69 (5.52)	reference	
120–129	2336	130 (5.57)	1.00 (0.71-1.42)	0.994
130–139	3220	201 (6.24)	1.21 (0.87-1.68)	0.252
140–149	2932	178 (6.07)	1.16 (0.83-1.62)	0.389
150–159	1900	151 (7.95)	1.45 (1.03-2.04)	0.035*
≥ 160	1669	143 (8.57)	1.69 (1.20-2.39)	0.003*
**DBP**				
< 80	5854	402 (6.87)	reference	
80-89	4992	320 (6.41)	0.95 (0.80-1.12)	0.510
90-99	2085	120 (5.76)	0.84 (0.67-1.06)	0.142
≥ 100	375	30 (8.00)	1.34 (0.91-1.98)	0.136

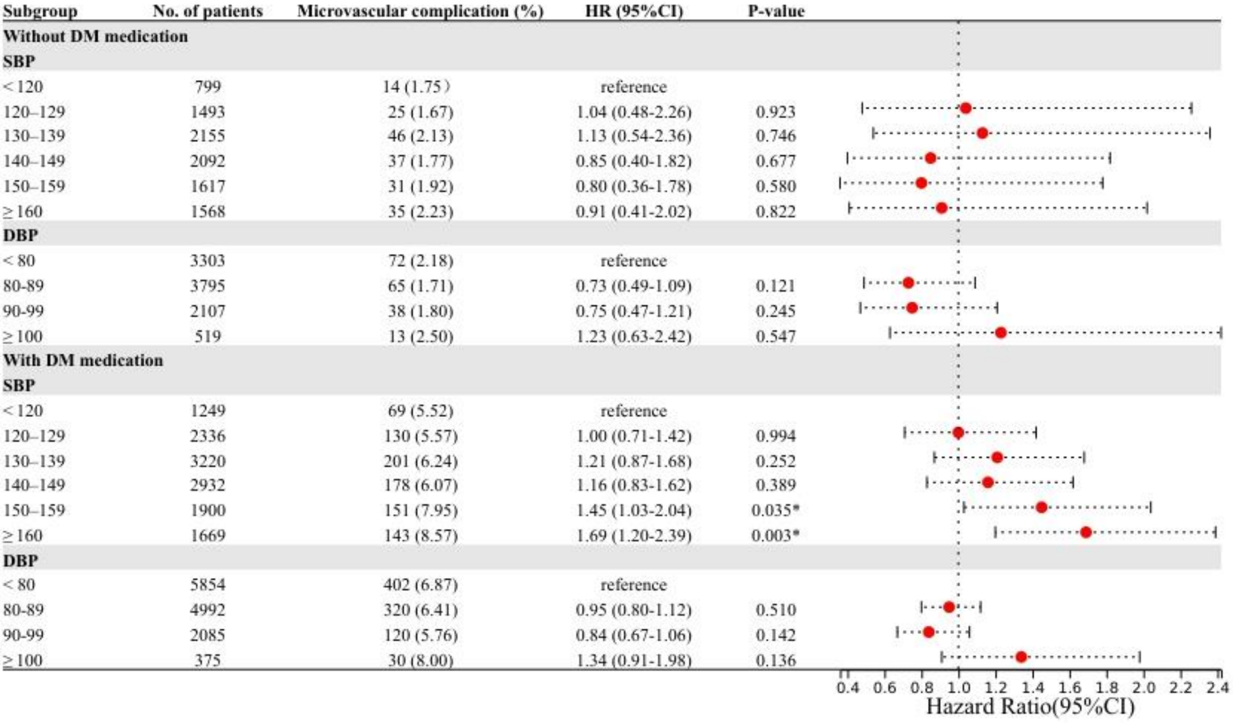

Figure S3. Blood pressure level and incident diabetic microvascular complications stratified by use of anti-hyperglycaemic medication

Cox regression models were used to estimate the hazard ratio (95% CI) for incident diabetic microvascular complications associated with blood pressure adjusted for age, sex, body mass index, ethnicity, Townsend index, smoking status, alcohol consumption, glycosylated haemoglobin, low-density lipoprotein cholesterol, duration of diabetes, anti-hypertensive medication, and estimated glomerular filtration rate.

DM, diabetes mellitus; SBP, systolic blood pressure; DBP, diastolic blood pressure; HR, hazard ratio; CI, confidence interval.

* Refers to statistically significant.

Subgroup	No. of patients	Microvascular complication (%)	HR (95%CI)	P-value
**Without antihypertensive medication**				
**SBP**				
< 120	1651	71 (4.30)	reference	
120–129	3046	121 (3.97)	0.86 (0.61-1.21)	0.377
130–139	4256	185 (4.35)	1.00 (0.73-1.39)	0.977
140–149	4002	173 (4.32)	1.02 (0.73-1.39)	0.922
150–159	2775	142 (5.12)	1.18 (0.84-1.66)	0.338
≥ 160	2519	136 (5.40)	1.32 (0.93-1.86)	0.118
**DBP**				
< 80	7114	360 (5.06)	reference	
80-89	6991	310 (4.43)	0.93 (0.78-1.10)	0.396
90-99	3410	123 (3.61)	0.79 (0.63-1.00)	0.053
≥ 100	734	35 (4.77)	1.36 (0.93-1.97)	0.112
**With antihypertensive medication**				
**SBP**				
< 120	397	12 (3.02)	reference	
120–129	783	34 (4.34)	2.37 (0.98-5.75)	0.057
130–139	1119	62 (5.54)	2.88 (1.23-6.76)	0.015*
140–149	1022	42 (4.11)	1.92 (0.79-4.66)	0.148
150–159	742	40 (5.39)	2.44 (1.00-5.93)	0.049*
≥ 160	718	42 (5.85)	3.45 (1.43-8.32)	0.006*
**DBP**				
< 80	2043	114 (5.58)	reference	
80-89	1796	75 (4.18)	0.82 (0.58-1.15)	0.244
90-99	782	35 (4.48)	1.01 (0.65-1.58)	0.955
≥ 100	160	8 (5.00)	1.21 (0.57-2.56)	0.617

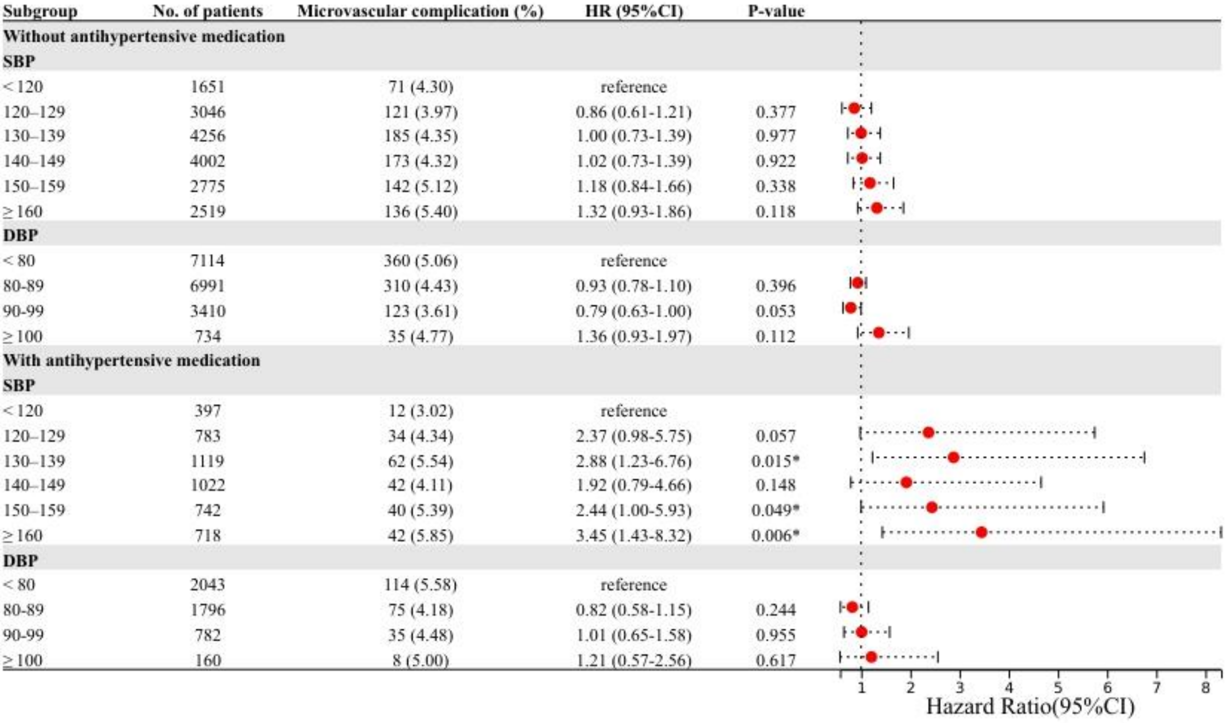

Figure S4. Blood pressure level and incident diabetic microvascular complications stratified by use of anti-hypertensive medication

Cox regression models were used to estimate the hazard ratio (95% CI) for incident diabetic microvascular complications associated with blood pressure adjusted for age, sex, body mass index, ethnicity, Townsend index, smoking status, alcohol consumption, glycosylated haemoglobin, low-density lipoprotein cholesterol, duration of diabetes, anti-hyperglycaemic medication, and estimated glomerular filtration rate.

SBP, systolic blood pressure; DBP, diastolic blood pressure; HR, hazard ratio; CI, confidence interval.

* Refers to statistically significant.

Subgroup	No. of patients	Microvascular complication (%)	HR (95%CI)	P-value
**Single medication or without**				
**SBP**				
< 120	1771	73 (4.12)	reference	
120–129	3289	129 (3.92)	0.90 (0.64-1.26)	0.548
130–139	4625	198 (4.28)	1.04 (0.76-1.43)	0.803
140–149	4367	179 (4.10)	1.00 (0.73-1.39)	0.985
150–159	3073	146 (4.75)	1.15 (0.82-1.61)	0.419
≥ 160	2809	142 (5.06)	1.26 (0.90-1.77)	0.183
**DBP**				
< 80	7749	381 (4.92)	reference	
80-89	7625	321 (4.21)	0.89 (0.75-1.06)	0.190
90-99	3758	127 (3.38)	0.77 (0.61-0.97)	0.026*
≥ 100	802	38 (4.74)	1.39 (0.97-2.00)	0.069
**Combination of antihypertensive and DM medication**				
**SBP**				
< 120	277	10 (3.61)	reference	
120–129	540	26 (4.81)	2.11 (0.79-5.67)	0.138
130–139	750	49 (6.53)	2.79 (1.09-7.11)	0.032*
140–149	657	36 (5.48)	2.05 (.078-5.41)	0.148
150–159	444	36 (8.11)	2.93 (1.11-7.70)	0.029*
≥ 160	428	36 (8.41)	3.82 (1.46-9.97)	0.006*
**DBP**				
< 80	1408	93 (6.61)	reference	
80-89	1162	64 (5.51)	0.90 (0.62-1.30)	0.563
90-99	434	31 (7.14)	1.24 (0.76-2.00)	0.386
≥ 100	92	5 (5.43)	1.16 (0.45-2.95)	0.763

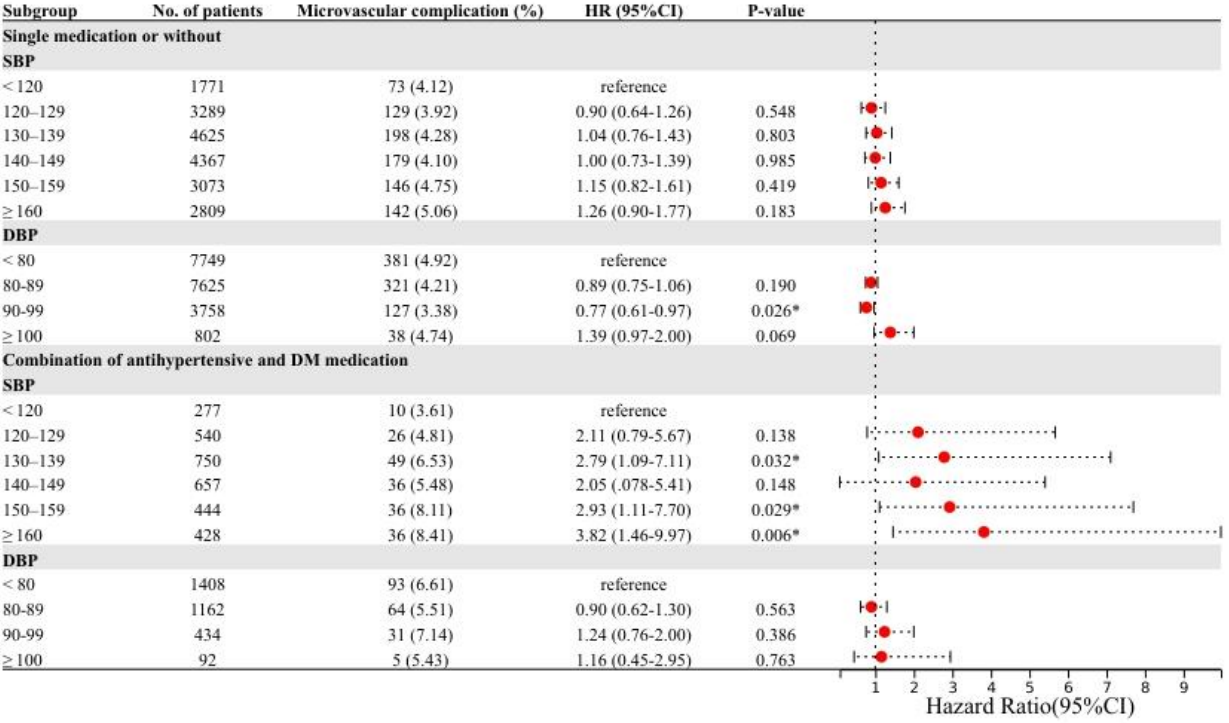

Figure S5. Blood pressure level and incident diabetic microvascular complications stratified by combinations of use of anti-hyperglycaemic and anti-hypertensive medication

Cox regression models were used to estimate the hazard ratio (95% CI) for incident diabetic microvascular complications associated with blood pressure adjusted for age, sex, body mass index, ethnicity, Townsend index, smoking status, alcohol consumption, glycosylated haemoglobin, low-density lipoprotein cholesterol, duration of diabetes, anti-hyperglycaemic medication, anti-hypertensive medication, and estimated glomerular filtration rate.

SBP, systolic blood pressure; DBP, diastolic blood pressure; DM, diabetes mellitus; HR, hazard ratio; CI, confidence interval.

* Refers to statistically significant.

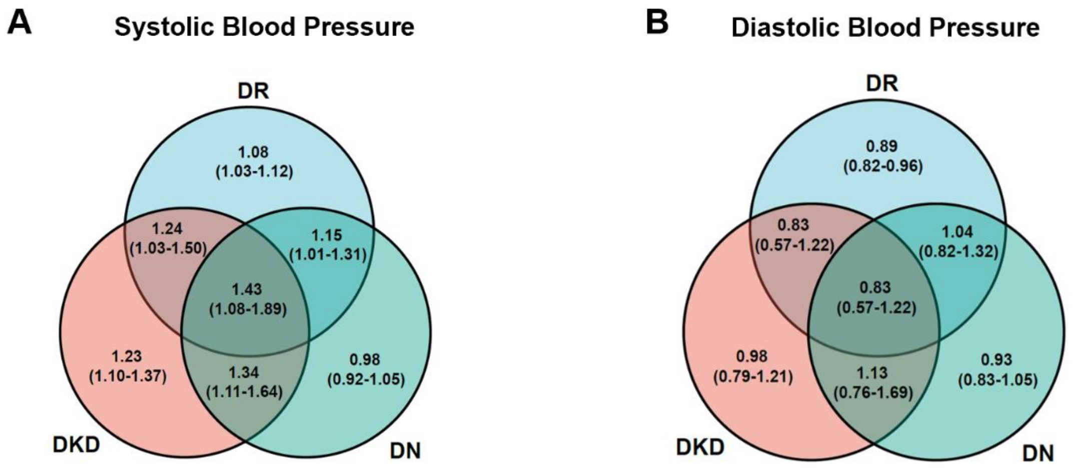

Figure S6. Cox proportional hazards models for diabetic microvascular complications: single and multiple complication models

HR and 95% CI adjusted for age and sex. DR, diabetic retinopathy; DKD, diabetic kidney disease; DN, diabetic neuropathy; HR, hazard ratio; CI, confidence interval.
